# Supplementary material for: Serological Screening for Middle East Respiratory Syndrome Coronavirus and Hepatitis E Virus in Camels in Kazakhstan
Source: Pathogens. 2022 Oct 24;11(11):1224. doi: 10.3390/pathogens11111224 (PMC9692571; doi:10.3390/pathogens11111224)
Supplement: Supplementary file 1 [file pathogens-11-01224-s001.zip › pathogens-1927089-supplementary.pdf]

Table S1. A semi-quantitative evaluation of the reactivity against HEV.

Sample #	Sample OD	Ratio	Positive Control OD
Turkestan 7	1.11	1.10	1.01
Turkestan 15	0.85	0.84	
Turkestan 33	0.77	0.77	
Turkestan 16	0.95	0.94	
Turkestan 9	1.01	1.01	
Turkestan 36	0.84	0.83	
Turkestan 19	1.33	1.32	
Turkestan 20	1.50	1.48	
Turkestan 5	1.26	1.25	
Turkestan 14	0.71	0.71	
Mangystau 11	0.78	0.72	1.08
Mangystau 20	1.21	1.12	
Jambyl 20	0.89	0.76	1.17
Jambyl 45	1.26	1.16	1.09
Jambyl 46	1.10	1.02	
Jambyl 47	1.05	0.97	
